# A Multisensor Fusion Method for Tool Condition Monitoring in Milling

**DOI:** 10.3390/s18113866

**Published:** 2018-11-10

**Authors:** Yuqing Zhou, Wei Xue

**Affiliations:** 1 College of Mechanical Engineering, Zhejiang University of Technology, Hangzhou 310014, China; zhouyq@wzu.edu.cn; 2 College of Mechanical and Electrical Engineering, Wenzhou University, Wenzhou 325035, China

**Keywords:** tool condition monitoring, milling process, multisensor fusion, kernel extreme learning machine, genetic algorithm

## Abstract

Tool fault diagnosis in numerical control (NC) machines plays a significant role in ensuring manufacturing quality. Tool condition monitoring (TCM) based on multisensors can provide more information related to tool condition, but it can also increase the risk that effective information is overwhelmed by redundant information. Thus, the method of obtaining the most effective feature information from multisensor signals is currently a hot topic. However, most of the current feature selection methods take into account the correlation between the feature parameters and the tool state and do not analyze the influence of feature parameters on prediction accuracy. In this paper, a multisensor global feature extraction method for TCM in the milling process is researched. Several statistical parameters in the time, frequency, and time–frequency (Wavelet packet transform) domains of multiple sensors are selected as an alternative parameter set. The monitoring model is executed by a Kernel-based extreme learning Machine (KELM), and a modified genetic algorithm (GA) is applied in order to search the optimal parameter combinations in a two-objective optimization model to achieve the highest prediction precision. The experimental results show that the proposed method outperforms the Pearson’s correlation coefficient (PCC) based, minimal redundancy and maximal relevance (mRMR) based, and Principal component analysis (PCA)-based feature selection methods.

## 1. Introduction

Milling is a very common and efficient cutting operation that uses a rotary milling cutter with one or more teeth to intermittently cut workpieces into flat surfaces, grooves, threads, and many complex geometric components. Highly efficient milling processes are suitable for mass production and have been used widely in the manufacturing industry. Tools are considered the pillars of the milling process [1], and tool breakage is a major cause of unscheduled stops in a machining environment. Tool breakage has negative direct (capital) and indirect (time loss) effects. The downtime of a milling machine due to tool failure accounts for 7–20% of total downtime [2,3], and the cost of tools and tool changes account for 3–12% of the total processing cost [4]. As the conditions of tools vary over time, the timeliness of detecting tool damage is critical, and it requires an appropriate tool replacement strategy. Conventionally, tool change strategies are subjective and regular and the time period is determined by the experience of the operator. While early replacement of a workable tool will waste tools and increase downtime, late replacement of a worn tool will result in lower quality workpieces and increased production costs [5]. Research [6,7] has determined that tools are typically only used for 50–80% of their effective lifespan. Thus, tool condition monitoring (TCM) has become an important challenge in the milling processes that schedules activities based on the result of condition measurements without interrupting normal machine operations [8]. TCM systems are developed in order to generate better surface quality and to extend tool life by diagnosing cutting tool deficiencies with appropriate signal processing and pattern recognition techniques, thereby reducing losses due to tool wear or failure. An accurate and reliable TCM system can reduce costs by 10–40% by reducing downtime and fully utilizing tools [7,9].

TCM in milling processes has been studied for over 30 years using two types of methods: Direct monitoring and indirect monitoring. Direct monitoring methods use optical equipment and machine vision technology to directly monitor the tool; for example, microscopes are used to capture tool images and evaluate the tool’s state with image analysis technology [10]. Direct methods are advantageous because they do not affect the machining process and they have high recognition accuracy under certain conditions. However, direct methods are not suitable for the manufacturing shop [11,12] because (1) the required equipment and the software are expensive, which could increase manufacturing costs, and (2) the recognition accuracy is easily disturbed by the cutting fluid and cutting chips. Therefore, indirect monitoring methods have been widely adopted. One or more sensors are used to measure a signal associated with the tool state, and the tool state is estimated by analyzing the measured signal. Compared with direct methods, indirect methods are cheaper and more adaptable to practical applications.

Indirect TCM is a data-driven method; it uses single or multiple sensors to monitor the milling process and synthesize information provided by the sensor signal to determine the best estimates for the tool state through a mechanism based on training [13]. Indirect TCM can be divided into two phases: Model training and online monitoring. The model training phase provides a training sample and then trains the monitoring model. The online monitoring phase monitors the milling process and estimates the cutting tool’s condition in real time. The model training phase consists of three modules: Sensor configuration, feature extraction, and monitoring model. The sensor configuration module provides an alternative sensory signal; the feature extraction module extracts features in the sensory signal that are related to the tool condition (wear, breakage, etc.). The monitoring model module builds a decision support model for online monitoring, and the online monitoring phase consists of two modules: Online sensory monitoring and decision-making. The sensor configuration from online monitoring is determined from the model training. If the sensors are not changed, then the configuration is the same in both online monitoring and the model training. If the sensors are changed, then the configuration from online monitoring will not consider sensors that are irrelevant to the tool condition.

Multisensors can provide more information related to the tool condition, but they increase the risk that effective information will be overwhelmed by redundant information. Thus, obtaining the most effective feature information from multisensor signals is currently a hot topic. In this paper, a multisensor global fusion method for TCM in the milling process is researched. Several statistical parameters in the time, frequency, and time–frequency domains of multiple sensors are selected as an alternative parameter set. The monitoring model is executed by the Kernel extreme learning machine (KELM), and the genetic algorithm (GA) is applied in order to search the optimal parameter combinations to achieve the highest prediction precision. The remainder of this paper is organized as follows: Section 2 reviews the literature of multisensor feature extraction in TCM for the milling process. Section 3 describes the theoretical framework and the learning algorithm of our proposed method. Section 4 describes an experiment with an open data set of the tool condition in order to compare the proposed method and several current feature extraction methods. Conclusions and suggestions for future work are given in Section 5.

## 2. Literature Review

Based on the literature, many sensors have been used in TCMs to obtain process signals [14,15,16,17], such as cutting force, vibration, current, and acoustic emission sensors. However, due to the uncertainty and limitations of single sensor monitoring, TCMs based on a single sensor cannot achieve good performance or robustness. Multiple sensor-based methods can enhance the richness of information that contains potential tool wear levels [18]. Although multisensor setups provide more redundant information, they can reduce the overall uncertainty of the measurement and improve the resolution and accuracy of the TCM system [4,19,20].

Therefore, multisensor monitoring has become a research trend. According to the statistics in Reference [21], the number of studies using multisensor-based methods has been gradually increasing for TCMs in the milling process. Recently, several studies found that the prediction accuracy of TCM is not positively related to the number of sensors and the number of feature parameters. The primary purpose of the feature extraction module is to extract feature parameters closely related to the tool state from the signals and to significantly reduce the dimensions of the original information. The result of the feature extraction module greatly affects the performance of the monitoring model. Too many feature parameters will greatly increase the model computation and affect the timeliness of online monitoring. In addition, irrelevant and redundant feature parameters have a negative impact on the performance of the monitoring model, and a few appropriate feature parameters can generate a more accurate and robust model [22,23].

The feature extraction methods for TCM in the milling process can be divided into four classifications, (1) time-domain-based method; (2) frequency-domain-based method; (3) wavelet analysis (time–frequency domain) based method; and (4) multidomain-based method. The methods based on the time domain extract feature information related to the tool state from the time dimension of the signal, including the time series analysis, several statistical parameters, and other information. The time series analysis includes Auto-Regression(AR), AutoRegressive Moving Average(ARMA), time domain averaging (TDA), and other information [3]. The statistical parameters include the root mean square error (RMSE), maximum/minimum, average, standard deviation, and kurtosis [24]. Frequency-domain-based methods extract feature information related to the tool state from the frequency dimension of the signal, including the frequency structure and harmonic component of the signal. These methods convert sensory signals from the time domain into the frequency domain with a fast Fourier transform and then extract feature parameters, such as the power spectrum, peak-to-peak amplitude, and tooth frequency [25]. The time-and-frequency-domain-based methods can only provide feature information from a single perspective, and both assume that the signal is stationary, which is not suitable for non-stationary signals in milling processes [26].

To overcome the shortcomings of the time-and-frequency-domain-based methods, a time–frequency analysis method based on a wavelet transform (WT) has been used for feature extraction in milling TCM. In WT-based methods, a discrete wavelet transform (DWT) [27], a continuous wavelet transform (CWT) [28,29], and a wavelet packet transform (WPT) [30] have been applied in order to extract a series of wavelet coefficients to reflect the tool state.

Multidomain-based methods select some parameters (such as the time domain, frequency domain, and time–frequency domain) to compose a candidate feature parameter set and apply certain feature selections or dimensional reduction methods to some feature parameters that are strongly related to the tool state. The advantage of multidomain methods is that they provide more candidate feature parameters related to the tool state and reduce the risk of losing important information, which is important for improving the performance of TCM. In the TCM model training phase, there is limited knowledge and experience to guide the selection of parameters. If the parameters closely related to the tool states are not selected for the candidate feature set, then the performance of the monitoring model could easily decrease. In the multidomain-based methods, although the number of feature parameters of the candidate feature set is clearly increased, the feature set is reduced to a low dimension by the feature selection or dimension reduction algorithm. Then, one needs to calculate the feature parameters after a dimension reduction in the online monitoring phase, which barely affects the operation speed of the model.

Currently, there are two kinds of multidomain feature extraction methods: Feature fusion and feature selection. Feature fusion methods obtain new parameters from preset sensor feature parameter sets by linear or nonlinear mapping, and the new feature parameters are used as the input of the monitoring model [31,32]. For example, Wang G.F. et al. [33] used the local preserving projection (LPP) algorithm to establish new fusion parameters and reduce the feature parameter dimension. Wang J. et al. [34] fused 54 feature parameters in the time-domain and the frequency-domain, as well as wavelet coefficients of 11 new feature parameters, using the kernel Principal component analysis (PCA) algorithm. The advantage of the feature fusion methods is they can use all preset feature parameters to provide information that is more comprehensive and that reduces feature dimensionality. However, in the online monitoring stage, all the sensors used in the training phase are needed and all the preset feature parameters are calculated, which could increase the maintenance cost and the computation for online monitoring. The feature selection methods select a few effective feature parameters from the preset candidate feature parameters. For example, Zhang et al. [22] selected 13 parameters significantly related to tool wear values using Pearson’s correlation coefficient (PCC) from 144 feature parameters extracted in the time, frequency, and time–frequency domains of multiple sensory signals. In Reference [35], the minimal redundancy and maximal relevance (mRMR) algorithm is utilized to select the most prominent features. Sohyung et al. [36] constructed 135 feature parameters in the time and frequency domains of multiple sensory signals and selected 25 feature parameters by employing the entropy correlation algorithm. Liu et al. [4] extracted 138 feature parameters (including time domain, frequency domain, and wavelet energy) as a candidate feature parameter set and used the fast correlation filter (FCBF) algorithm to establish the smallest redundant feature set, including 19 feature parameters. Feature selection methods can reduce the number of input parameters of the monitoring model by screening out the characteristic parameters that have a strong correlation with the tool state, but they also reduce the computation of the online monitoring stage. However, most of the current feature selection methods take into account the correlation between the feature parameters and the tool state and do not analyze the influence of the feature parameters on prediction accuracy. The parameters related most strongly to the tool state cannot generate the highest prediction accuracy. It is necessary to find out the global optimal combination of the sensor feature parameters for prediction accuracy.

## 3. Theoretical Framework

### 3.1. The Framework of TCM

**Figure 1:**
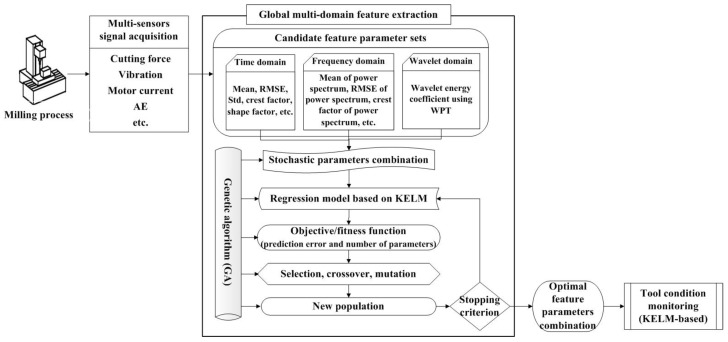
Framework of the proposed tool condition monitoring method. AE: Acoustic emission, RMSE: Root mean square error, WPT: Wavelet packet transform, KELM: Kernel extreme learning machine.

In this paper, a multisensor global fusion method for TCM is constructed, whose structure is shown in Figure 1. The operation of this method is composed of three steps. The first step is multisensor signal acquisition. Dynamic signals from multiple sensors (such as force, vibration, and current) are collected in order to depict the characteristic of the milling process. The second step is global feature extraction. A few statistical parameters in the time, frequency, and time–frequency domains of multiple sensors are extracted as candidate parameters. The KELM is applied as the monitoring model and the base of prediction precision. Two objective functions include the prediction error and the number of parameters, and the optimal sensor feature parameters are sought in order to realize the smallest prediction error and the least number of parameters through a modified GA. The last step is to monitor the tool state and judge the tool wear value using KELM, in which the inputs are the optimal sensor feature parameters.

### 3.2. Kernel Extreme Learning Machine

An ELM first proposed by Huang et al. [37,38] is proposed for the single hidden-layer feed forward neural networks. A Kernel extreme learning machine (KELM) is an application of ELM with a kernel function that has the advantage of a fast learning speed and high efficiency compared to other learning algorithms, such as Support vector machine(SVM), back propagation neural networks(BPNN), and least square SVM(LS–SVM) [39].

The KELM theory tends to reach not only the smallest training error, but also the smallest norm of the output weights. This objective function for the regression task can be expressed as follows:

(1) $$\underset{\gamma ,\varepsilon }{\min}\;\frac{1}{2}{\left\|\beta \right\|}_{F}^{2}+\frac{C}{2}\sum_{i=1}^{n}{\left\|\varepsilon_{i}\right\|}_{}^{2}\;s.t.\;\varepsilon_{i}=y_{i}-\beta^{T}f(x_{i}),\;\forall i,$$

where (*X*,*Y*) = {(*x_i_*,*y_i_*), *i* = 1,…, *n*} is the training sample set, n is the number of training samples, *f*(*x_i_*) = {*f*(*x_i_*_1_),…, *f*(*x_i__L_*)} is the hidden-layer output vector with respect to *x_i_*, *f*(•) is a form of feature mapping that maps the input data from the original dimension space to the l-dimensional hidden-layer feature space, *β =* {*β*_1_,…, *β_L_*}^T^ is the vector of the output weights between the hidden layer of the *L* nodes and the output node, *ε_i_* is the training error of the *i*-th training sample, and *C* is the regularization parameter that trades off the norm of output weights and training errors. ||•||*_F_* is the Frobenius norm.

According to Reference [37], the optimal *βˆ* that minimizes Equation (1) can be efficiently solved as:

(2) $$\hat{\beta }=\Phi^{T}{\left(\frac{I}{C}+\Phi \Phi^{T}\right)}^{-1}Y,$$

where *F* is the hidden-layer output matrix, $F=\left[\begin{array}{c} f(x_{1})\\ M\\ f(x_{n}) \end{array}\right]=\left[\begin{array}{ccc} f(x_{11}) & \Lambda & f(x_{1L})\\ M & \Lambda & M\\ f(x_{n1}) & \Lambda & f(x_{nL}) \end{array}\right]$, *I* is an identity matrix, and $Y={\left\{y_{1},\Lambda \;y_{n}\right\}}^{T}$ is the dependent value vector in the training samples. The prediction score *y_x_*’ on test point *x* is determined by:

(3) $$y_{x}^{\prime }=\varphi (x)\hat{\beta }.$$

Since the signal of the multisensors in TCM is high-dimension, nonlinear, and heterogeneous, the feature mapping φ(⋅) is unknown. It is necessary to define a kernel matrix for ELM using Mercer’s conditions:

(4) $$\Psi =\Phi \Phi^{T}=\{\phi_{ij}\},\;\phi_{ij}=\varphi (x_{i})\varphi (x_{j})=k(x_{i},x_{j}).$$

Then, the prediction score on test point x in Equation (3) can be rewritten as:

(5) $$y_{x}^{\prime }=\varphi (x)\Phi^{T}{\left(\frac{I}{C}+\Phi \Phi^{T}\right)}^{-1}Y={\left[\begin{array}{c} k(x,x_{1})\\ M\\ k(x,x_{n}) \end{array}\right]}^{T}{\left(\frac{I}{C}+\Psi \right)}^{-1}Y.$$

In this context, similar to SVM, the feature mapping φ(⋅) does not need to be known, and a common kernel function can be used, e.g., a Gaussion kernel, linear kernel, or polynomial kernel. In addition, the dimensionality *L* of the feature space (number of hidden nodes) does not need to be given. It is noteworthy that the kernel matrix Ψ is only related to the input data *x_i_* and the number of training samples [39].

### 3.3. Global Feature Extraction

Different from most of the current feature selection methods that consider the correlation (linear/nonlinear) between the feature parameters and the tool state, the prediction accuracy of the TCM method was taken into account in this paper.

In this paper, we want to reach not only the smallest prediction error, but also the lowest number of parameters. These two objective functions can be expressed as follows:

(6) $$\min\;\frac{1}{m}\sqrt{\sum_{t=1}^{m}{\left(Y_{Xt}-Y_{Xt}^{\prime }\right)}^{2}}\;\&\;\min\;\sum_{q=1}^{Q}\delta_{q},$$

where *Y_Xt_* is the dependent value with respect to *X_t_*, *Y’_Xt_* is the predicted value with respect to *X_t_* using the KELM, *δ_q_* denotes the state of the *q*-th feature parameter, and *δ_q_* = 1 indicates that the *q*-th feature parameter is included in the input set of KELM regression model; otherwise, *δ_q_* = 0, and *Q* is the number of candidate feature parameters. The first objective function calculates the RMSE of the selected data, and the second objective function calculates the number of parameters used in the KELM regression model.

**Figure 2:**
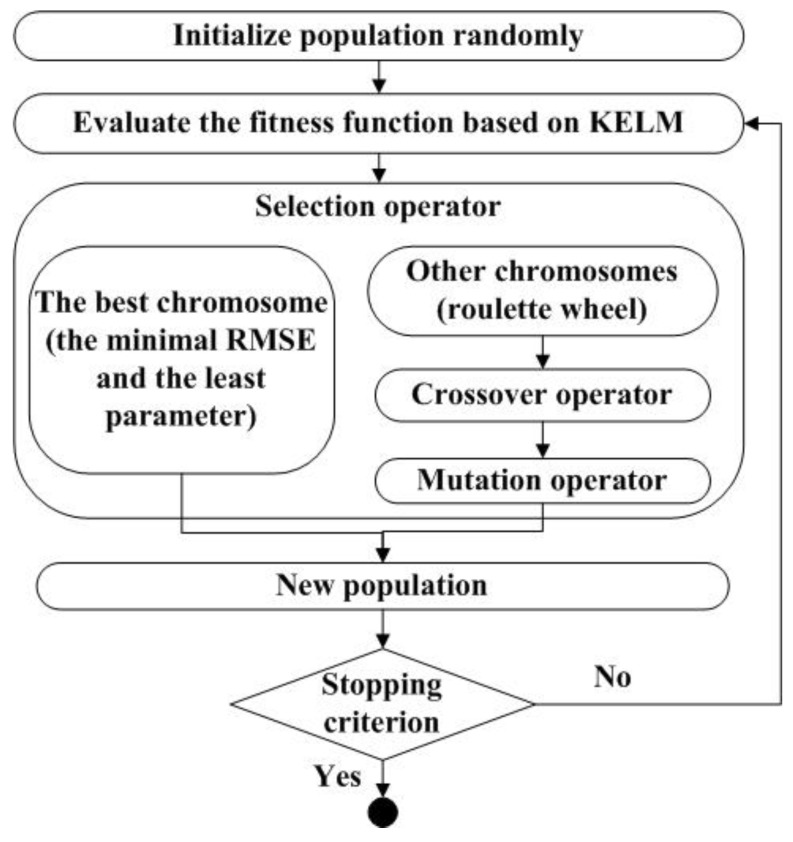
The process of global feature extraction algorithm based genetic algorithm (GA).

Mathematically, the problem of Equation (6) is formulated as a combinatorial optimization problem. Here, the function used for optimization is the generalization performance of the predictive model, represented by the error of a training data set, and the design variables are the inclusion (1) or the exclusion (0) of the candidate parameters. Therefore, the two-objective optimization problem in Equation (6) can be transformed into a single objective optimization, in which the second objective is embedded in the optimization with the first objective, while remaining the best solution with the fewest parameters in each optimization iteration. An exhaustive selection of candidate feature parameters would evaluate many different combinations (=2Q). This process becomes impracticable when Q is large. The GA algorithm is an intelligent optimization method for function optimization based on the mechanics of natural genetics and biological evolution, which is capable of solving the global optimization of complex problems [40]. In this paper, GA was used to optimize Equation (6), and the global feature extraction algorithm-based GA is shown in Figure 2.

In Figure 2, the fitness function is the RMSE with the training set using KELM. Obviously, a high RMSE indicates a low fitness, and those individuals with greater fitness will have a greater probability of being selected for recombination. The selection operator divides into two parts. The first part is the best chromosome that has a minimal RMSE and the lowest number of parameters, and that can solve the second objective in Equation (6). The other part selects the individuals according to their fitness level using the roulette wheel method. The crossover operator and the mutation operator recombine the selected individuals in order to generate a new population with a preset crossover rate *Pc* (the chance of crossover being applied to a chromosome) and mutation rate *Pm* (the chance of a chromosome being mutated). Finally, the iterations are completed when the number of iterations reaches the preset maximum iteration value.

## 4. Experiments

### 4.1. Descriptions of Datasets

**Figure 3:**
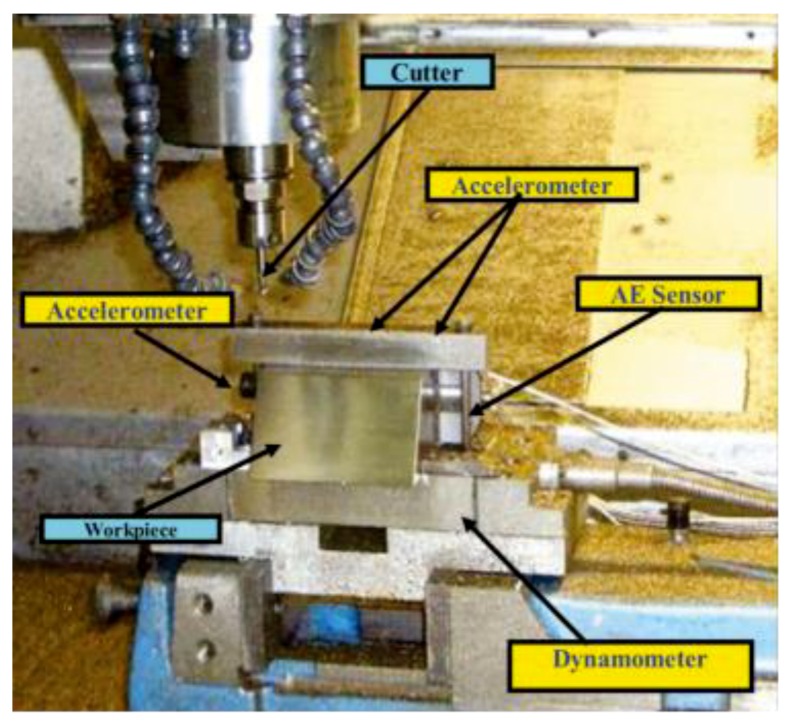
The experimental setup.

In the present work, the TCM prediction problem reported in the 2010 Prognostics and Health Management (PHM) data challenge [41] was used to verify the performance of the proposed method. The experimental setup is shown in Figure 3 [42], and this dataset was collected from a high-speed Computerized numerical control (CNC) milling machine (Type RFM 760 from Roders Tech Co. Ltd in Germany) under dry milling operations. The operation parameters in this experiment are shown in Table 1.

The workpiece surface was machined to have a slope with 60° to accommodate the 2-flute ball nose cutter. A Kistler quartz 3-component platform dynamometer was mounted between the workpiece and the machining table to measure the cutting forces in the form of charges, which were then converted to voltages by the Kistler charge amplifier. Three Kistler piezoelectric accelerometers were mounted on the workpiece to measure the machine tool vibrations of the cutting process in the X, Y, and Z directions, respectively. A Kistler acoustic emission (AE) sensor was mounted on the workpiece to monitor the high frequency stress wave generated by the cutting process. Therefore, the sensory data consisted of seven channels: The force in three directions, the vibration in three directions, and the AE–RMS. A DAQ NI PCI1200 was adopted to perform in-process measurements, including the force and vibration in three directions (x, y, and z), with a continuous sampling frequency of 50 KHz during the tool wear test. The corresponding flank wear of each individual flute was measured offline using a LEICA MZ12 microscope after finishing each surface. Finally, three individual cutter records named C1, C4, and C6 were selected as our dataset, and each record contained 315 data samples. Two tests, C1 and C4, were used as the training sample, and C6 was used as the testing set.

### 4.2. Candidate Parameter Sets

To overcome the drawback of features in a single domain, which lose some useful information related to the tool condition, in this study, the multidomain features of multisensor signal were extracted in the time, frequency, and time–frequency domains. According to previous papers [34,43,44] and our experimental studies [45], a few dimensional and dimensionless statistical feature parameters in the time, frequency, and time–frequency (wavelet) domains were chosen.

As shown in Table 2, nine statistical feature parameters related to the tool state from the time dimension of the sensor signal were extracted as candidate parameters, including three dimensional features—the average value, the root mean square, and the standard deviation—and six dimensionless features—the crest factor, the shape factor, the waveform, the kurtosis factor, the skewness factor, and the margin factor.

As shown in Table 3, eight statistical feature parameters related to the tool state from the frequency domain of the sensor signal were extracted as candidate parameters, including two dimensional features—the mean and the root mean square of the power spectrum—and six dimensionless features—the crest factor of the power spectrum, the modified equivalent bandwidth, the high–low ratio of the power spectrum, the stabilization ratio, the skewness of the bandpower, and the kurtosis of the bandpower.

**Figure 4:**
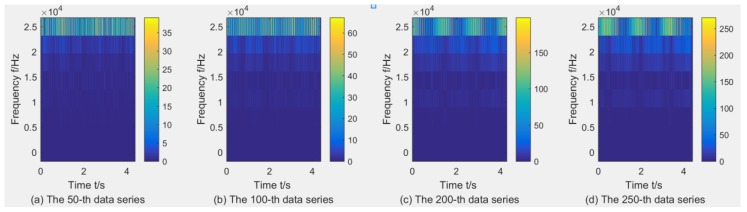
Wavelet time–frequency diagrams for the X-dimension force in the C1 training sample through WPT using db1

**Figure 5:**
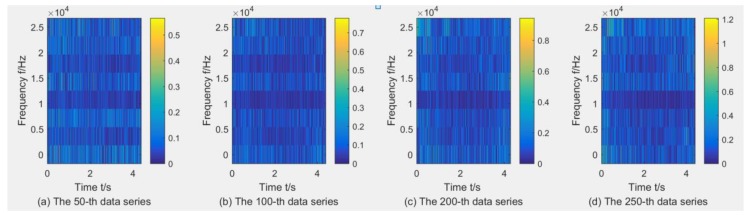
Wavelet time–frequency diagrams for the X-dimension Vibration in the C1 training sample through WPT using db1.

**Figure 6:**
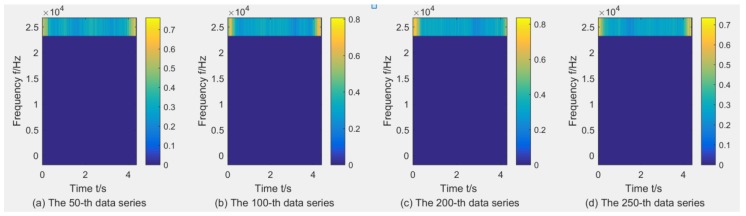
Wavelet time–frequency diagrams for the AE in the C1 training sample through WPT using db1.

In the time–frequency domain, Wavelet transform (WT) can be used to extract candidate feature parameters. The Wavelet packet transform (WPT) conducts a multilevel band division over the entire signal band, which not only inherits the advantages of the good time–frequency localization from the WT, but it also further decomposes the high-frequency band to increase the frequency resolution [4,22,34]. Thus, the WPT was applied in order to extract the time–frequency domain features in this paper, and the wavelet energy feature is the energy of a 3-level wavelet packet decomposition using db1, which corresponds to the wavelet coefficient with a higher energy that is related to the characteristic frequency of the machine [46]. The average energies for each frequency band of the signal were calculated with the following equation:

(7) $$E_{j}=\frac{1}{n}\sum_{k=1}^{n}{\left(d_{j,k}^{}\right)}^{2}=\frac{1}{n}\sum_{k=1}^{n}{\left(\int w_{j,k}(t)x(t)dt\right)}^{2},$$

where $d_{j,k}^{}(j=1,2,\Lambda ,2^{L};k=1,2,\Lambda ,n)$ denotes the wavelet packet coefficients of signal *x*(*t*) and *w_j,k_*(*t*) are the wavelet packets localized at 2*^j^k* in the scale 2*^j^*, and *L* is the level of the WPT (here, *L* = 3). Taking the C1 sample as an example to show the wavelet time–frequency diagrams through WPT using db1, due to limited space, three wavelet time–frequency diagrams with the X-dimension force, X-dimension Vibration, and AE are listed in Figure 4, Figure 5 and Figure 6. It can be found that the maximal wavelet coefficients of the X-dimension force in the C1 training sample are enhanced with the increase of tool wear, while that of other two sensors showed no obvious trend of change.

Finally, there were (9 + 8 + 8) × 7 = 175 features to be extracted from the seven sensor channels in the time, frequency, and time–frequency domains.

### 4.3. Results and Discussion

**Figure 7:**
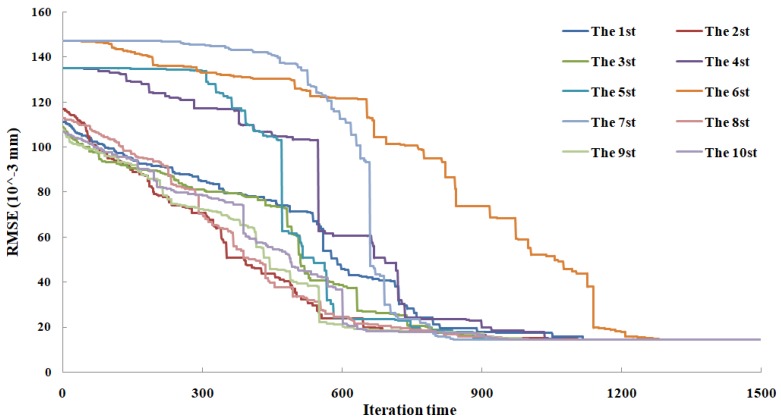
The optimal process of the objected function.

In the proposed GA–KELM-based global feature extraction algorithm, the input parameters used are summarized in Table 4. The optimal process of the objected function was run 10 times, and the iterations of optimal solutions are shown in Figure 7. It can be shown that the optimal result was obtained when the iteration was run about 1000 times.

**Figure 8:**
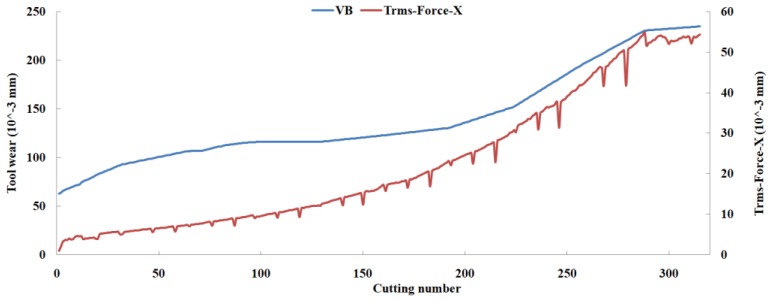
The scatter diagram between *T_rms_* in X-dimension Force and tool wear value.

**Figure 9:**
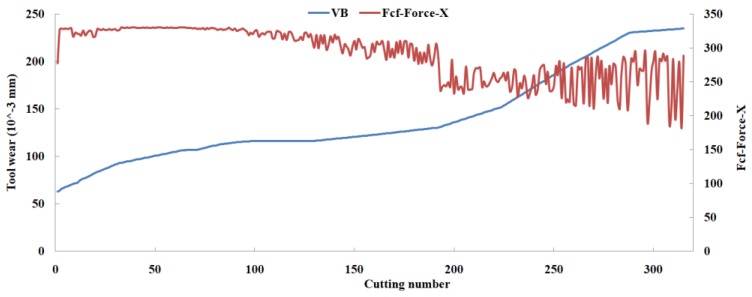
The scatter diagram between *F_cf_* in X-dimension Force and tool wear value.

**Figure 10:**
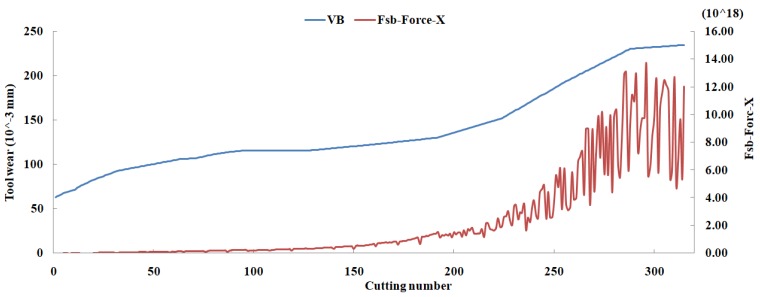
The scatter diagram between *F_sb_* in X-dimension Force and tool wear value.

**Figure 11:**
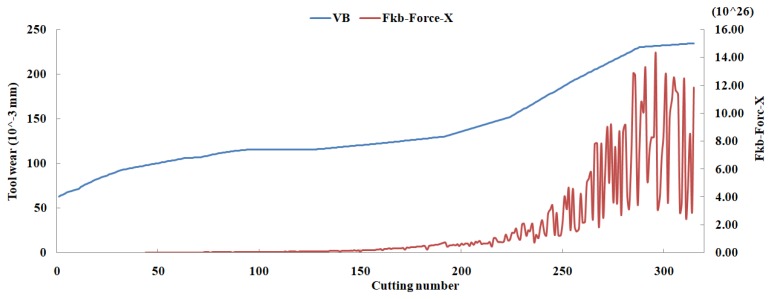
The scatter diagram between *F_kb_* in X-dimension Force and tool wear value.

**Figure 12:**
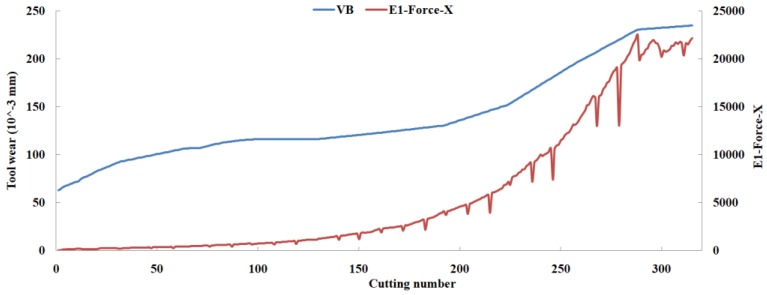
The scatter diagram between *E*_1_ in X-dimension Force and tool wear value.

**Figure 13:**
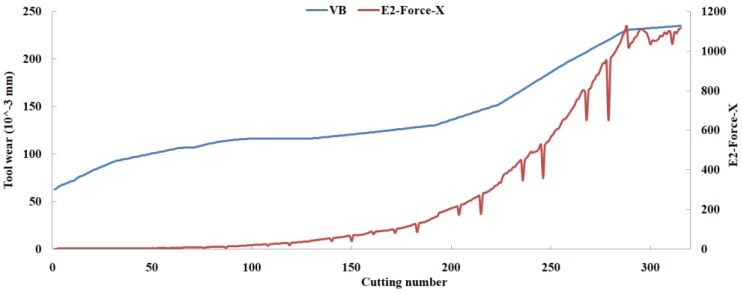
The scatter diagram between *E*_2_ in X-dimension Force and tool wear value.

**Figure 14:**
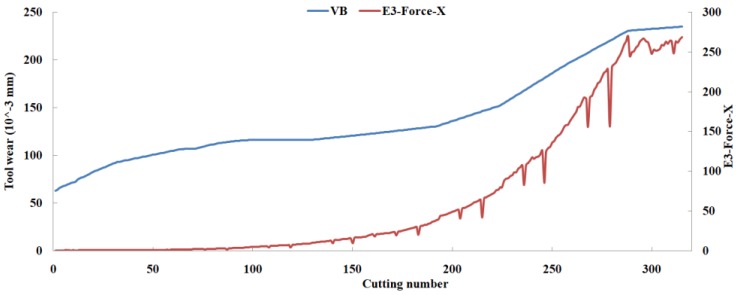
The scatter diagram between *E*_3_ in X-dimension Force and tool wear value.

**Figure 15:**
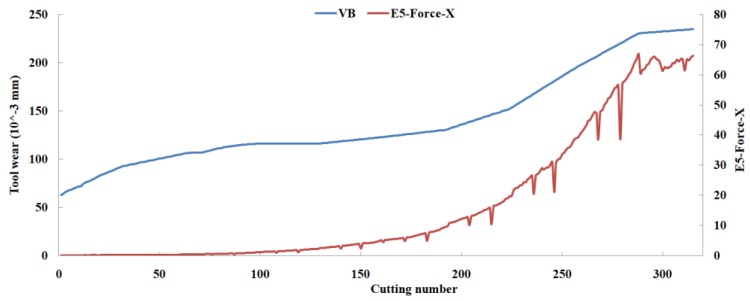
The scatter diagram between *E*_5_ in X-dimension Force and tool wear value.

**Figure 16:**
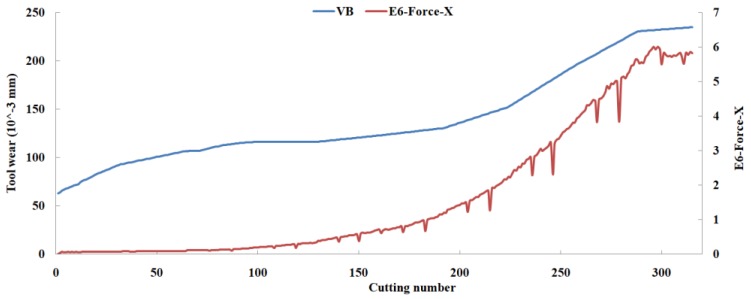
The scatter diagram between *E*_6_ in X-dimension Force and tool wear value.

**Figure 17:**
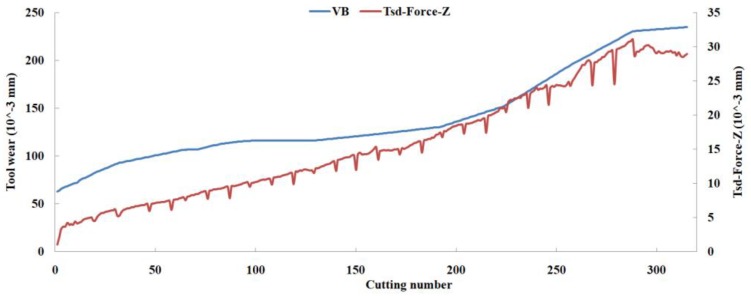
The scatter diagram between *T_sd_* in Z-dimension Force and tool wear value.

**Figure 18:**
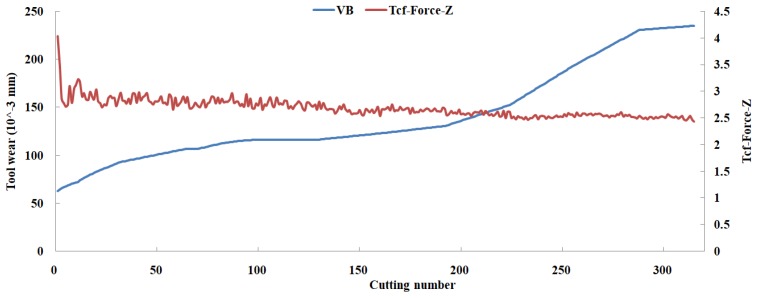
The scatter diagram between *T_cf_* in Z-dimension Force and tool wear value.

Eleven feature parameters were selected as the optimal feature parameter set, which involved two sensor channels, *T_rms_*, *F_cf_*, *F_sb_*, *F_kb_*, *E*_1_, *E*_2_, *E*_3_, *E*_5_, and *E*_6_ in the X-dimension Force, and *T_sd_*, and *T_cf_* in the Z-dimension Force. Figure 8, Figure 9, Figure 10, Figure 11, Figure 12, Figure 13, Figure 14, Figure 15, Figure 16, Figure 17 and Figure 18 show the scatter diagrams between these feature parameters selected and tool wear value (VB) with the C6 testing set. It can be seen that four feature parameters have no obvious correlation with the tool state, that is, *F_cf_*, *F_sb_*, *F_kb_* in the X-dimension Force, and *T_cf_* in the Z-dimension Force. This demonstrates that the parameters related most strongly to the tool state could not obtain the highest prediction accuracy, that is to say, not every parameter is strongly related to tool state in the optimal parameters corresponding to prediction accuracy.

**Figure 19:**
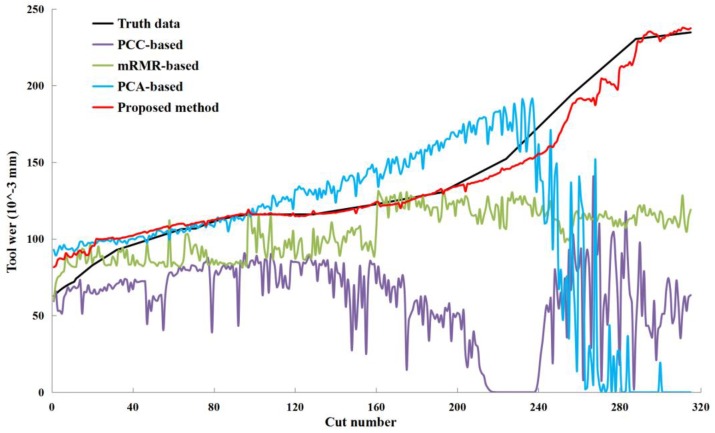
Predicted tool wear values of several methods with the C6 testing set.

The predicted tool wear values with the C6 testing set are shown in Figure 19. It was found that the overall error of the predicted tool wear was small, and the proposed method could reveal the trend of the tool wear state well. In addition, three benchmark methods, PCC-based, mRMR-based, and PCA-based, were compared with the proposed method. The PCC [24,47,48], mRMR [34,35], and PCA [31,32] methods are often used to extract feature parameters or principal components in TCM according to linear or nonlinear correlation. In the PCC-based method, these feature parameters of the correlation coefficient with tool wear values above 0.8 were selected as the inputs of the KELM in this study, and 33 parameters were retained in the final features. In the mRMR-based method, 19 parameters were selected in the final features by considering the maximum relevance and the minimum redundancy criterion simultaneously. In the PCA-based method, principal components were obtained through solving the eigenvalues of the correlation matrix of training data. The cumulative contribution rate of the first 14 principal components reached 90.23%, and then the first 14 principal components were selected as the inputs of the KELM. The predicted tool wear values of the three methods with the C6 testing set are shown in Figure 16, and several performance indexes (RMSE and R^2^ with the truth data, the number of selected parameters, and the number of sensor channels involved) of the four methods are shown in Table 5. It can be shown that the proposed method outperforms the other three methods in terms of prediction accuracy and the number of selected parameters.

## 5. Conclusions

The present study proposed a multisensor global fusion method for TCM in the milling process. Several statistical parameters in the time, frequency, and time–frequency domains of multiple sensors were selected as an alternative parameter set. The GA was applied in order to search the optimal parameter combinations to achieve the highest prediction precision, while the monitoring model was executed by a KELM. The experimental results show that the proposed method outperforms the PCC-based and mRMR-based methods. The RMSE and R^2^ values for the truth data, the number of selected parameters, and the number of sensor channels of the proposed method were better than those of the other two methods.

## Figures and Tables

**Table 1:** Operation parameters in the experiment.

Operation Parameter	Value
CNC machine	Roders Tech RFM 760
Workpiece material	Inconel 718 (Jet engines)
Cutter	3-flute ball nose
Spindle speed	10,400 RPM
Feed rate	1555 mm/min
Y depth of cut (radial)	0.125 mm
Z depth of cut (axial)	0.2 mm
Number of sensors	5
Number of sensor channels	7
Sampling data	50 KHz/channel

**Table 2:** Nine time domain statistical parameters.

Domain	Indexes	Formula
Time	Average Value *T_avg_*	$T_{avg}=\sum_{j=1}^{n}x_{j}/n$
	Root mean square *T_rms_*	$T_{rms}=\sqrt{\sum_{j=1}^{n}x_{j}^{2}/n}$
	Standard Deviation *T_sd_*	$T_{sd}=\frac{1}{n-1}\sqrt{\sum_{j=1}^{n}{\left(x_{j}-T_{avg}\right)}^{2}}$
	Crest Factor *T_cf_*	$T_{cf}=\max\{\left|x_{j}\right|\}/T_{rms}$
	Shape factor *T_sf_*	$T_{sf}=T_{rms}/T_{avg}$
	Waveform *T_wf_*	$T_{wf}=n\cdot T_{rms}/\left(\sum_{j=1}^{n}\left|x_{j}\right|\right)$
	Kurtosis Factor *T_ku_*	$T_{ku}=(\sum_{j=1}^{n}{\left(x_{j}-T_{avg}\right)}^{4})/\left(n\cdot T_{sd}^{4}\right)-3$
	Skewness Factor *T_sk_*	$T_{sk}=(\sum_{j=1}^{n}{\left(x_{j}-T_{avg}\right)}^{3})/\left(n\cdot T_{sd}^{3}\right)$
	Margin factor *T_mf_*	$T_{sk}=n^{2}\cdot \max\{\left|x_{j}\right|\}/{\left(\sum_{j=1}^{n}\sqrt{\left|x_{j}\right|}\right)}^{2}$

**Table 3:** Eight frequency domain statistical parameters ^1^.

Domain	Indexes	Formula
Frequency	Mean of power spectrum *F_mps_*	$F_{mps}=\sum_{j=1}^{n}P_{j}/n$
	Root mean square of power spectrum *F_rms_*	$F_{rms}=\sqrt{\sum_{j=1}^{n}P_{j}^{2}/n}$
	Crest factor of power spectrum *F_cf_*	$F_{cf}=\max\{\left|P_{i}\right|\}/F_{rms}$
	Modified equivalent bandwidth *F_meb_*	$F_{meb}=\sqrt{\left(\sum_{j=1}^{n}{\left(f_{j}-\bar{f}\right)}^{2}P_{j}\right)/\left(\sum_{j=1}^{n}P_{j}\right)}$
	High–low ratio of power spectrum *F_hlps_*	$F_{hlps}=\left(\sum_{j=n/4}^{n/2}P_{i}\right)/\left(\sum_{j=1}^{n/4}P_{i}\right)$
	Stabilization ratio *F_sr_*	$F_{sr}=\left(\sum_{j=1}^{n}f_{j}^{2}P_{i}\right)/\left(\sqrt{\sum_{j=1}^{n}P_{i}}\sqrt{\sum_{j=1}^{n}f_{j}^{4}P_{i}}\right)$
	Skewness of bandpower *F_sb_*	$F_{sb}=\left(\sum_{j=1}^{n}{\left(P_{i}-F_{mps}\right)}^{3}\right)/{\left(\sum_{j=1}^{n}{\left(P_{i}-F_{mps}\right)}^{2}\right)}^{\frac{3}{4}}$
	Kurtosis of bandpower *F_kb_*	$F_{sb}=n\cdot \left(\sum_{j=1}^{n}{\left(P_{i}-F_{mps}\right)}^{4}\right)/\left(\sum_{j=1}^{n}{\left(P_{i}-F_{mps}\right)}^{2}\right)$

^1^*f_i_* is the frequency signal with *x_i_* by FFT, *P_i_* is the power spectrum of *f_i_*, $\bar{f}=\sqrt{\sum_{i=1}^{n}f_{i}^{}/n}$.

**Table 4:** Input parameters in the proposed GA–KELM-based algorithm.

Parameters	Value
Size of the population for every generation	50
Crossover rate *Pc*	0.6
Mutation rate *Pm*	0.05
Number of iterations	1500
Regularization parameter *C*	6
Kernel function	Gaussion kernel
Hyperparameter of kernel	2

**Table 5:** Several performance indexes of three methods on the testing set.

Methods	RMSE	R^2^	Number of Selected Parameters	Number of Sensor Channels Involved
The PCC-based method	98.339	−0.198	33	5
The PCA-based method	94.665	−0.5768	175	7
The mRMR-based method	53.268	0.598	19	5
The proposed method	24.711	0.988	11	2
